# Anemia Profile in Elite Israeli Olympic-Level Athletes—Is Screening Necessary?

**DOI:** 10.3390/nu17243827

**Published:** 2025-12-06

**Authors:** Ori Abulafia, Alon Eliakim, Tahel Shilat, Yoram Epstein, Dan Nemet

**Affiliations:** 1 The Elite Sport Department of Israel, Wingate Institute, Netanya 4290200, Israel; oriabulafia@mail.tau.ac.il; 2 School of Public health, Gray Faculty of Medical and Health Sciences, Tel Aviv University, Tel Aviv 6997801, Israel; 3 Department of Pediatrics, Meir Medical Center, Kfar-Saba 4428164, Israel; eliakim.alon@clalit.org.il; 4 School of Medicine, Gray Faculty of Medical and Health Sciences, Tel Aviv University, Tel Aviv 6997801, Israel; hlrinst@tauex.tau.ac.il; 5 Edelson School of Medicine, Ariel University, Ariel 5545173, Israel; tahel.shilat@mail.huji.ac.il; 6 Faculty of Health Sciences, Department of Sport therapy, Ono Academic College, Kiryat Ono 3133801, Israel; 7 Olympic Committee of Israel, Tel Aviv 6948206, Israel

**Keywords:** anemia, iron metabolism, elite athlete, screening

## Abstract

**Background:** Screening blood tests are often collected from elite athletes in an effort to optimize performance. The purpose of the current study was to evaluate the yield of screening for anemia and anemia-related factors in elite athletes entering the Israeli Olympic team. **Methods:** We investigated hemoglobin levels, red blood cell count and indices and serum levels of iron, transferrin, ferritin, B12 and folic acid in 407 members of the Israeli Olympic team (179 females, 228 males) upon joining the team. **Results:** Forty-four (10.8%) athletes had abnormally lower Hb level (8.9% females and 12.3% males). Forty-two athletes (10.3%) had low RBC concentration (9.5% females and 11% males). Twenty-one athletes (5.2%) had low iron levels (7.9% in males and 1.7% in females) and only 14 athletes (7 males) had low ferritin levels (3.4%). Fourteen female athletes (7.8%) had ferritin levels of less than 20 ng/mL, and 43 (24%) had levels of less than 30 ng/mL. There were no cases of both anemia and low ferritin levels together. Twenty-five athletes (6.1%) had low levels of folic acid with higher prevalence (7%) in males. Only five athletes (1.2%) had low levels of vitamin B12, while 29 (7.1%) had levels higher than normal. None of them had abnormal Hb. In a multiple regression analysis, combat sports had significantly lower Hb levels. **Conclusions:** The yield of anemia screening and especially anemia-related biochemical abnormalities in adult elite athletes may be relatively low. Clearly, anemia should not be missed in the elite athlete, yet, if there are no signs or symptoms, Hb levels are close to normal and dilution is diagnosed, further frequent biochemical evaluation may be unnecessary.

## 1. Introduction

In sports medicine, athletes regularly undergo blood tests to ensure a proper health status. Although there is no consensus on blood test screening protocols, complete blood count (CBC) is considered one of the most critical laboratory exams to define the health status of an athlete [1]. CBC can reveal low concentrations of hemoglobin (Hb) and anemia, which requires further biochemical analysis to define and treat the cause. Low serum levels of iron and ferritin are often the underlying cause of anemia.

Iron is a fundamental mineral used by the body for numerous processes. Besides being an essential nutrient in the synthesis of heme, which is important to the hemoglobin structure, iron is also involved in energy metabolism within the electron transport chain, DNA synthesis, oxidative phosphorylation in mitochondria and ATP production [2].

The most common cause of anemia is iron deficiency. Iron is a transition metal and has multiple functions in more than 180 biochemical reactions in the human body, including oxygen-binding proteins, critical in physical performance [2,3]. When we consume iron, the absorbed iron is stored as ferritin in the cytoplasm of enterocytes. To export the iron to the plasma, the iron carried out by ferroportin (FPN) on the basolateral surface of the enterocytes. In the plasma, iron is bound to transferrin and transported to the liver where it is stored as ferritin or transferred to the iron consuming tissues. The classic diagnostic triad of iron deficiency anemia is low hemoglobin, microcytosis and low serum ferritin concentration. Blood ferritin concentrations correlate well with bone marrow iron, and are considered a sensitive indicator of body iron stores. Decreased ferritin level is usually the first indicator of depleted iron stores, and reduced transferrin saturation and anemia appear only at later stages of iron deficiency [4].

Clearly, as these processes are imperative to support athletic endeavors, the provision, utilization and storage of iron is extremely important in athletes. Iron deficiency is a widely reported issue in athletic population, with a documented prevalence reported ranging between 15 and 35% in female and ~3–11% in male athletes [5]. Apart from iron deficiency, other causes for anemia in athletes include other nutritional deficiencies [vitamin B12, folic acid (FA)], hemodilution and redistribution, and hemolysis or bleeding (menstrual) [2].

We previously demonstrated a relatively high (13%) incidence of depleted iron stores in 114 members of the Israeli Olympic team [4]. We also reported high prevalence of non-anemic iron deficiency (NAID) (~30%) in adolescent male and female athletes from the Israeli National Center for Children Gifted in Sports [6]. Due to these results, efforts were made to increase the nutritional awareness of the athletes, the coaches and accompanying staff. Upon joining the Olympic team, a nutritional evaluation, including blood tests are performed, and nutritional consultations are offered on a regular basis to all members of the Israeli Olympic team.

The aim of the present study was to evaluate the prevalence of anemia, and to report the serum levels of iron, transferrin, ferritin, B12 and folic acid in elite athletes entering the Israeli Olympic team, and to explore the effects of gender and types of sports on these variables.

## 2. Materials and Methods

### 2.1. Participants

The study population included male and female elite athletes from the Israeli Olympic team between the years 2002 and 2024 (96.8% of samples were collected after 2016). Blood tests were taken as part of routine evaluation of the Israeli Olympic team members. For the current study, we selected only the *first test* of each athlete upon entering the Olympic team, and we report 407 baseline tests (179 females, 228 males, age 13 to 39 years) Overall. The study was approved by the Meir Medical Center Institutional Review Board.

The Israeli National Olympic team includes athletes ranked at the top 16 in European/World Championship or equal international competitions at senior and junior levels. Exclusion criteria include any chronic disease that may affect blood tests results. This study does not include blood samples containing less than 40 biomarker results (non-screening tests) or disease-related tests (blood samples contain unique biomarkers) to avoid tests ordered due to specific clinical reasons. In addition, any blood samples collected on dates other than the predetermined screening test dates were excluded from the study for reasons similar to those discussed above.

### 2.2. Blood Sampling Collection and Analysis

Blood samples were collected by the medical team at the Ribstein Center for Sports Medicine Sciences and Research at the Wingate Institute early in the morning, following 12 h of fasting (water was allowed ad libitum) and at least 12 h from the last workout. Upon collection, samples were stored in temporary refrigerators and transported immediately to Meir Medical Center (MMC) labs for analysis. The transport is performed in an insulated cool box under the Ministry of Health’s guidelines.

Blood samples were analyzed at the MMC labs within 24 h after collection. The systems used for analyzing the samples were the ADVIA 2120i (Siemens Healthineers, Munich, Germany). The results were then uploaded to a private cloud safe and stored in an athlete management system “Smartabase” (Fusion Sport Pty Ltd., Brisbane, Australia) as approved by Clalit Health Services and the Israeli Ministry of Health.

### 2.3. Data Analysis

Sport types were grouped based on similar physiological characteristics. Athletes not falling into any predefined category were labeled as “Other”. Sport types with less than 10 athletes were also labeled as “Other”. The original sport types alongside their assigned groups are presented below.

### 2.4. Type of Sport

In order to compare the different types of sports, we divided the athletes into several categories, in the following order [7]:

- Anaerobic: power lifting and anaerobic track and field.
- Endurance: triathlon, cycling, long distance running.
- Endurance strength: artistic gymnastic, climbing.
- Fencing: fencing.
- Combat: judo, taekwondo, wrestling, boxing.
- Rhythmic Gymnastics
- Sailing: sailing, surfing.
- Swimming: swimming.
- Artistic swimming: artistic swimming.
- Other: alpine skiing, skateboard, figure skating, short-track speed skating, basketball 3 × 3, handball, badminton, beach volleyball, tennis, breakdance, modern pentathlon, soccer, archery, baseball, golf, shooting, table tennis, kayaks, and rowing.

For each blood marker, the following statistical measures were computed for both genders: mean, standard deviation (STD), percentage of high abnormalities, percentage of low abnormalities, and total percentage of abnormalities. Linear regression was utilized to assess the influence of gender, sport type, and age on each blood marker. In cases where the assumptions of linear regression were not met, robust regression techniques were employed to ensure the validity of the analysis. For blood markers exhibiting more than 8% abnormal results, logistic regression was performed using the same set of variables to explore potential predictors of abnormalities. We included only sport types that were markedly different (either very low or very high) in the descriptive statistics as dependent variables. These selected sports were then collectively compared against all other sport types. Statistical significance is considered as *p* ≤ 0.05. All data were analyzed through Python 3.7 (Python Software Foundation, www.python.org, Wilmington, DE, USA).

## 3. Results

Numbers of female and male participants in each sport category are presented in Table 1.

The baseline Hb, Ht, RBC count, RBC indices, and transferrin (*n* = 407, 179 females, 228 males) are presented in Table 2.

Overall, 44 (10.8%) athletes had abnormally lower Hb level (8.9% females and 12.3% males). Fifty-five athletes had low Ht levels (13.4%), 38 males (16.7%) and 17 females (9.5%). Forty-two athletes (10.3%) had low RBC concentration (9.5% females and 11% males). Twenty-five athletes had both low Hb and low RBC concentration. MCHC was higher than the population upper normal range in 37 athletes (9%), more so in males (28/228, 12.3%).

The results of the baseline Iron, ferritin, transferrin, B12 and folic acid levels are presented in Table 3.

Overall, males were found to have low abnormal iron values compared to females (*p* = 0.03). Twenty-one athletes (5.2%) had low iron levels (7.9% in males and 1.7% in females) and only 14 athletes (7 males) had low ferritin levels (3.4%). Fourteen female athletes (7.8%) had ferritin levels of less than 20 ng/mL, and 43 (24%) had levels of less than 30 ng/mL.

Altogether, fifty athletes (12.3% of all screenings) had a serum ferritin less than 30 ng/mL. Only one female athlete had both low iron, low ferritin (17 ng/mL) and low Hb, and this athlete also had microcytic RBCs. When ferritin levels were set at the lower normal population range (10 ng/mL for females and 30 ng/mL for males), five athletes (three males, two females) had both low iron and low ferritin. There were no cases of both anemia and low ferritin (≤10 ng/mL for females and ≤30 ng/mL for males) levels together. Twenty athletes had low transferrin levels (4.9%).

Twenty-five athletes (6.1%) had low levels of Folic acid with higher prevalence (7%) in males. Only five athletes (1.2%) had low levels of vitamin B12, while 29 (7.1%) had levels higher than normal. None of them had low iron levels or abnormal Hb.

### Multiple Regression Analysis

Logistic regression analysis (Table 4) demonstrated that combat sport athletes had a significantly higher likelihood of exhibiting abnormally low Hb levels. Combat sport athletes tended to have lower RBC concentration, yet this reached statistical significance only in males (*p* = 0.01).

## 4. Discussion

The present study demonstrates a relatively high incidence (10.8%) of low hemoglobin levels among elite Olympic athletes entering the Olympic team. Interestingly, the prevalence of anemia was higher in male athletes, especially in combat sports. The low Hb level was mostly associated with low Ht but not with either low iron or ferritin levels or low FA and vitamin B12 levels.

The main rationale for including routine hematological assessment during periodic health examination of the athlete is based on the higher-than-expected prevalence of decreased iron stores in athletes, particularly female athletes [8]. In females, in addition to inadequate dietary intake, menses are a major cause of iron loss. In our elite athlete cohort, the prevalence of anemia was lower in female athletes compared to males. This is encouraging since our previous report demonstrated depleted iron stores in Olympic female athletes [4], and efforts were made to monitor athletes in order to decrease the prevalence of iron deficiency and anemia in female athletes. Even though the incidence of anemia in our males lies within the reported 5–15% of the anemic male athlete cohorts [2,5], it is clearly a call to ensure proper monitoring and treatment in our male athletic population as well.

Some of our athletes were found to have anemia with low iron and “normal” ferritin concentrations. This does not follow the classic findings of iron deficiency anemia. Exercise leads to a significant inflammatory response [9], with increasing levels of inflammation-responsive cytokines such as interleukin 6 (IL-6), a major regulator of hepcidin expression [10]. Hepcidin is a key regulator of the entry of iron into the circulation. The magnitude of the inflammatory response and the upregulation of hepcidin depends on the type, intensity and duration of the exercise [5]. Since ferritin also acts as an acute-phase protein, it should be considered that sporting activities might increase acute-phase reactants. For example, in the case of ultramarathons, ferritin may be twice as high as the pre-race value and returns to baseline only 6 days after the race [11,12]. Thus, when evaluating anemia in athletes, especially during periods of heavy training or competition, the classical triad of iron deficiency anemia diagnosis may not be sensitive enough to represent the “true” body iron stores. Evaluating anemia following sufficient rest is advisable, yet not always possible, and clearly, adding measurement of serum iron and/or inflammatory markers (hs-CRP, hepcidin) to the usually measured ferritin in the cases of anemia workup may be needed.

The exact recommended ferritin level in athletes and when iron replacement should be started is debated and has been reported at various ferritin levels [13]. When applying the population recommended cut-off levels of ferritin (20 ng/mL) to our female athlete cohort, only 7.8% athletes had low ferritin levels. Yet, when the minimal cut-off value is set to 30 ng/mL, as recommended by the Swiss Society of Sports Medicine [3], the Australian Institute of Sports and others [13], a quarter of our female athletes had insufficient concentrations of serum ferritin. This condition is termed nonanemic iron deficiency (NAID), and even though it mostly does not affect performance, it does suggest that these athletes should be further monitored closely for iron status to decrease their risk for developing overt anemia.

With this said, twenty-five out of our forty-four anemic athletes (56%) were found to have low RBC concentration with low Ht, normal MCV and MCH, and normal iron, ferritin, FA and vitamin B12 levels. This may suggest that many of the cases of low hemoglobin in our cohort are caused by hemodilution or redistribution when increased Hb mass is outpaced by plasma expansion, leading to ‘pseudo-anemia’ [14]. Dilutional pseudo-anemia cannot be ruled out only by a venous blood examination, but in clinical practice serial measurements of the basic blood examination may be helpful to demonstrate variations in the hematocrit. Other volume markers may also assist in the diagnosis [3].

Interestingly, we found significantly lower Hb levels in combat sports (judo, taekwondo, wrestling and boxing). One third of anemic athletes (5/15) had low iron levels, while eight athletes (8/15) had low RBC concentrations. The reasons for this finding in our large cohort are unknown and require further evaluation and monitoring. Some studies do suggest that anemia may be more prevalent in young judokas [15] compared to other sports, while Drid et al. [16] reported that weight reduction in male judokas may be associated with increased prevalence of pseudo-anemia.

A significant number of athletes had increased B12 levels (7.1%); none of them had low iron levels. In Israel, many of the iron supplements also contain B12, some with a relatively high dose. Unfortunately, we do not have data on supplement consumption, but we can only speculate that some of the higher-than-normal-range B12 levels are related to supplementation.

While our study included a relatively large number of elite athletes (407 Olympic level athletes), its main limitation is the relatively small number of athletes in each sport category. To overcome this, we gathered similar sports into groups, and as a result most categories are sufficient in numbers. In addition, the study examined anemia profile when athletes first joined the Olympic team. Athletes may join the Olympic team during different parts of the competitive season. This might be a limiting factor, even though values are reported not to change significantly during the season [17]. To overcome this, all tests were performed after at least 12 h of rest, in the morning and in a fasted state to minimize variability. Another limitation is the lack of assessment or adjustment for iron or vitamin supplementation; the relatively low prevalence of anemia among female athletes may suggest nutritional intervention.

## 5. Conclusions

In summary, based on our results, and consistent with some previous reports [14,18,19], we suggest that the yield of anemia screening and especially anemia-related biochemical screening in adult elite Olympic level athletes may be relatively low. Many of the low-Hb cases found in our cohort were associated with low Ht and RBC concentrations, suggesting pseudo-anemia, and their further biochemical evaluation yielded no benefit to the athletes. With this said, anemia should clearly not be missed in the elite athlete; yet if there are no signs or symptoms, Hb levels are close to the normal range, and dilution is diagnosed, further frequent biochemical evaluation may be unnecessary.

## Figures and Tables

**Table 1:** Numbers and age of male and female participants by sports.

Sport	Sex	Number	Age, Years
Anaerobic	Female	12	24.2 (6.1)
	Male	13	22.8 (6.5)
Endurance	Female	12	23.4 (3.3)
	Male	39	24.1 (4.4)
Endurance strength	Female	5	22.4 (5.5)
	Male	19	21.6 (3.5)
Fencing	Female	14	18.3 (2.3)
	Male	17	20.6 (4.7)
Combat	Female	35	21.5 (4)
	Male	41	21.6 (2.6)
Artistic Gymnastics	Female	33	16.9 (1.9)
Sailing	Female	21	20.2 (3.2)
	Male	18	20.5 (2.6)
Swimming	Female	13	19.6 (4.3)
	Male	42	20.7 (3.4)
Artistic swimming	Female	18	18.2 (2.6)
Other	Female	16	22.7 (4.9)
	Male	39	23.1 (3.9)

**Table 2:** Baseline Hb, RBC and its indices, and transferrin (*n* = 407, Female 179).

	Gender	Normal Range	Mean (std)	High (n)	Low (n)	Normal (n)	High (%)	Low (%)	Total Abnormal (%)
HB g/dL	Female	12.0–16.0	12.95 (0.76)	0	16	163	0	8.9	8.9
	Male	13.5–17.5	14.56 (0.91)	0	28	200	0	12.3	12.3
Ht %	Female	36.0–44.0	39.27 (2.42)	0	17	162	0	9.5	9.5
	Male	41.0–50.0	43.53 (2.58)	0	38	190	0	16.7	16.7
RBC M/µL	Female	4.0–5.5	4.37 (0.29)	0	17	162	0	9.5	9.5
	Male	4.5–5.9	4.93 (0.34)	0	25	203	0	11	11
MCV fL	Female	80.0–100.0	90.03 (4.03)	2	1	176	1.1	0.6	1.7
	Male		88.46 (3.63)	0	1	227	0	0.4	0.4
MCH pg	Female	26.0–34.0	29.68 (1.54)	3	3	173	1.7	1.7	3.4
	Male		29.57 (1.39)	1	4	223	0.4	1.8	2.2
MCHC g/dL	Female	30.0–34.5	32.96 (0.97)	9	1	169	5	0.6	5.6
	Male		33.44 (0.95)	28	0	200	12.3	0	12.3

**Table 3:** Iron, ferritin, transferrin, folic acid and B12 levels (*n* = 407, female 179).

Biomarker	Gender	Normal Range	Mean (std)	High (n)	Low (n)	Normal (n)	High (%)	Low (%)	Total Abnormal (%)
Iron µg/dL	Female	37.0–145.0	98.93 (36.78)	16	3	160	8.9	1.7	10.6
	Male	59.0–158.0	106.86 (37.89)	19	18	191	8.3	7.9	16.2
Ferritin ng/mL	Female	10.0–150.0	52.46 (30.76)	1	7	171	0.6	3.9	4.4
	Male	30.0–400.0	114.07 (68.46)	1	7	220	0.4	3.1	3.5
Transferrin mg/dL	Female	200.0–360.0	259.71 (36.96)	2	5	172	1.1	2.8	3.9
	Male		244.66 (33.87)	0	15	213	0	6.6	6.6
Folic Acid ng/mL	Female	3.9–26.8	9.63 (3.90)	0	9	170	0	5	5
	Male		8.3 (3.72)	0	16	212	0	7	7
Vitamin B12 ng/L	Female	180.0–940.0	582.99 (239.69)	13	2	164	7.3	1.1	8.4
	Male		569.05 (227.01)	16	3	209	7	1.3	8.3

**Table 4:** Logistic regression analysis (Hb).

Variable	β Coefficient	OR (95% CI)	*p*-Value
Age	−0.02	0.98 (0.91–1.07)	0.70
Gender (male)	0.41	1.51 (0.77–2.92)	0.23
Combat sport	0.96	2.61 (1.32–5.18)	<0.001

## Data Availability

The data presented in this study are available on request from the corresponding author due to privacy reasons.
